# Rapid and Sensitive Detection of Rutin in Food Based on Nitrogen-Doped Carbon Quantum Dots as Fluorescent Probe

**DOI:** 10.3390/molecules27248834

**Published:** 2022-12-13

**Authors:** Yue Huang, Xiaojing Si, Mei Han, Chen Bai

**Affiliations:** Department of Food Science, Shanghai Business School, Shanghai 200235, China

**Keywords:** nitrogen-doped carbon quantum dots, hydrothermal synthesis, fluorescence probe, rutin

## Abstract

The aim of this study was to establish a rapid detection method of rutin in food based on nitrogen-doped carbon quantum dots (N-CDs) as the fluorescent probe. N-CDs were prepared via a single-step hydrothermal process using citric acid as the carbon source and thiourea as the nitrogen source. The optical properties of N-CDs were characterized using an electron transmission microscope, X-ray diffractometer, Fourier-transform infrared spectrometer, and nanoparticle size potential analyzer. The UV/Vis absorption property and fluorescence intensity of N-CDs were also characterized using the respective spectroscopy techniques. On this basis, the optimal conditions for the detection of rutin by N-CDs fluorescent probes were also explored. The synthesized N-CDs were amorphous carbon structures with good water solubility and optical properties, and the quantum yield was 24.1%. In phosphate-buffered solution at pH = 7.0, Rutin had a strong fluorescence-quenching effect on N-CDs, and the method showed good linearity (R^2^ = 0.9996) when the concentration of Rutin was in the range of 0.1–400 μg/mL, with a detection limit of 0.033 μg/mL. The spiked recoveries in black buckwheat tea and wolfberry were in the range of 93.98–104.92%, the relative standard deviations (RSD) were in the range of 0.35–4.11%. The proposed method is simple, rapid, and sensitive, and it can be used for the rapid determination of rutin in food.

## 1. Introduction

**Figure 1:**
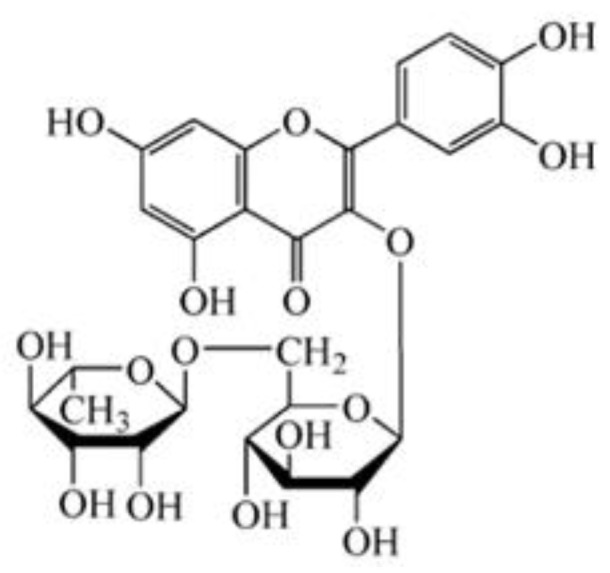
Chemical structural formula of rutin.

Rutin is referred to as vitamin P and rutinoside, with the chemical formula 3,3′,4′,5,7-pentahydroxyflavone-3-rutinoside and the chemical structure shown in Figure 1. It belongs to the class of naturally biological products known as flavonoids. Rutin is mainly derived from and exists in medicinal and food plants, such as *Sophora japonica*, buckwheat, wolfberry, *Astragalus membranaceus*, and *Pueraria*, as well as vegetables, such as asparagus, tomato, and cucumber, and fruits such as jujube, apricots, oranges, grapefruit, lemons, and oranges [1,2]. A good volume of research has revealed a variety of biological activities of rutin, including antidiabetic, antioxidant, antimicrobial, antifungal, antiallergic, anticancer, neuroprotective, and cardioprotective potential [3,4,5,6,7,8]. With the improvement in general public health, as well as people’s lifestyle and living standard, rutin is attracting increasing attention on account of the multitude of its activities. The rutin content varies in different foods due to different origins or collection times. Meanwhile, the content derived from different plant parts in food is also different. For example, the rutin content in *Sophora japonica* is 10–28% [9]. The rutin content in the shell of Tartary buckwheat is the highest, followed by its embryo and bran, while the content in the endosperm is the lowest [10,11]. In the past two decades, the biological activities of rutin have been increasingly recognized, and the extraction technology and preparation methods have also made great progress. Therefore, the determination of the content of the active ingredient in rutin is essential for its utilization in food and drugs. In addition, quick detection and screening of foods with high rutin content have great demand on the market.

At present, liquid chromatography [12], capillary electrophoresis [13], electrochemical analysis [14], chemiluminescence [15], and fluorescence [16] have been effectively used for the determination of rutin. These techniques can furnish accurate results and are widely applied in the detection of rutin. Carbon quantum dots (CDs), as a novel class of photoluminescence nanomaterial, are known for their small size and distinctive physical and fluorescence characteristics. In recent years, CDs have been the focal point of interest from worldwide researchers, and they have found successful applications in bioanalytical detection, labeling, imaging, catalytic sensing, and other areas. The fluorescence intensity of CDs quenched or augmented within a specified concentration range is related to the concentration of the particular drug. Hence, CDs can be employed as a fluorescence probe for drug detection [17]. In analytical detection, CDs are a good substitute for conventional fluorescent chemicals due to their favorable fluorescence performance, good water solubility, and low toxicity. They also offer immense potential for detecting food components and pollutants [18]. Additionally, CDs with heteroatom doping can successfully modify the internal structure and electron distribution of their quantum dots to increase their stability, as well as the yield of fluorescence quantum and photoelectric performance. As a result, it might be a good idea to develop a straightforward and efficient fluorescence detection method for rapid analysis of rutin content in food based on CDs.

In the preparation process of CDs, atomic doping (nitrogen, phosphorus, boron, etc.) can not only improve CDs, but also provide active sites, thereby broadening their potential applications in analytical detection and other fields. Nitrogen is abundant in nature and simple to obtain. It is a frequently used doping element to enhance the optical and material properties of CDs due to its five valence electrons and atomic size comparable to that of carbon [19]. In addition, nitrogen can provide excess *n* electrons, such that the Fermi level can be moved up and the optical properties can be changed [20,21]. So far, various nitrogen-containing compounds have been explored as precursors for nitrogen doping of CDs.

In this study, nitrogen-doped CDs (N-CDs) were synthesized using a one-step hydrothermal process, and their fluorescence was quenched by reacting with rutin. It is possible to quickly and sensitively identify the presence of rutin in food using N-CDs as the fluorescence probe since the degree of quenching is related to the concentration of rutin within a specified range. This study establishes a feasible fluorescence detection method and offers the technical basis for the quick detection of rutin in food, in addition to providing a certain theoretical base for the advancement of flavonoid detection.

## 2. Results and Discussion

### 2.1. Characterization of N-CDs

**Figure 2:**
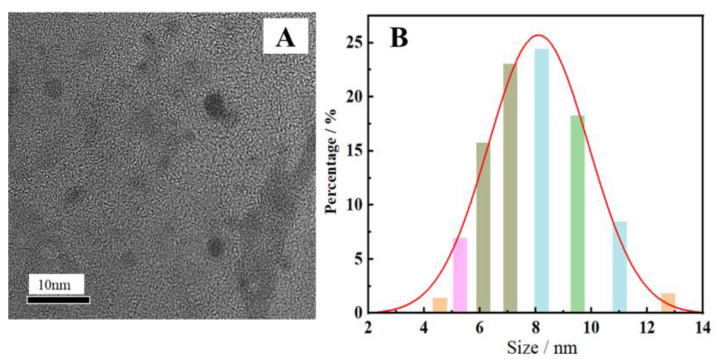
TEM image (**A**) and particle size distribution (**B**) of the synthesized N-CDs.

The morphology and particle size of the synthesized N-CDs, as determined by a TEM and nanoscale potential analyzer, are presented in Figure 2. Figure 2A illustrates that the CDs were spherical with high dispersion and uniform size. It is evident from Figure 2B that the particle size ranged from 6 to 9 nm, satisfying the size specifications of CDs. The elemental analysis diagram of N-CDs from an energy-dispersive spectrometer (EDS) is shown in Table 1. The results indicate that the N-CDs were made up of the components C, N, O, and S, with the corresponding mass ratios of 88.46%, 5.04%, 5.93%, and 0.5%, respectively.

**Figure 3:**
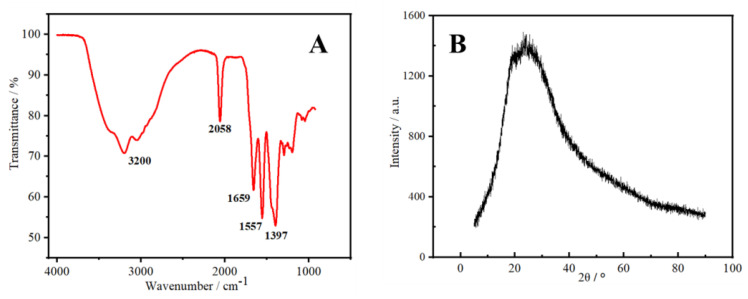
FT−IR spectrum (**A**) and XRD pattern of the synthesized N-CDs (**B**).

In Figure 3A, the peak of N-CDs in the range of 3200 cm^−1^ was assigned to the stretching vibration of the overlapping O–H and N–H bands [22]. The asymmetric stretching vibration peak of the triple-bond region and the cumulative double bond at 2058 cm^−1^ may have been caused by O=C=O. The strong absorption at 1659 cm^−1^ was assigned to the stretching vibration peak of C=O, indicating the existence of the carbonyl group [23]. The peak at 1557 cm^−1^ could be attributed to the bending vibration peak of N–H, whereas the peak at 1397 cm^−1^ corresponded to the stretching vibration of C–N [24]. This analysis suggested that the surface of N-CDs was rich in amino, hydroxyl, and carbonyl groups. The existence of these oxygen-containing groups also implies that N-CDs had good water solubility. Figure 3B shows the XRD pattern of N-CDs. N-CDs gave rise to a wide absorption peak at 23.9°, which is a characteristic peak indicating an amorphous carbon structure [25]. The above characterization results indicate that the preparation of N-CDs material via a one-step hydrothermal method was efficient.

### 2.2. Optical Properties of N-CDs

**Figure 4:**
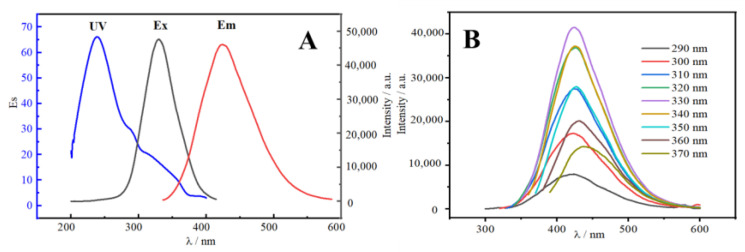
(**A**) UV/Vis absorption (UV), excitation (E_x_), and emission (E_m_) spectra of N-CDs. (**B**) Emission spectra at various wavelengths of excitation (290–370 nm).

The UV/Vis absorption (UV), fluorescence excitation (E_x_), and fluorescence emission (E_m_) were measured. The UV, E_x_, and E_m_ spectra of N-CDs all exhibited a tendency to grow first and subsequently reducing, as illustrated in Figure 4A. The E_x_ and E_m_ spectra both exhibited good symmetry. Due to the creation of the C=C skeleton between N-CDs and the π–π* transition in the sp^2^ of N-CDs, the N-CDs exhibited a clear UV absorption peak at 240 nm [26]. For N-CDs, 330 and 425 nm were the ideal excitation and emission wavelengths, respectively. In Figure 4B, the emission spectrum of N-CDs displays a gradual red-shift in excitation wavelength from 290 to 370 nm, with the fluorescence intensity first increasing and then decreasing. This reflects the clear excitation wavelength dependency of CDs, which is created by the p–π* transition following the excitation of the C=C unsaturated link in N-CDs [27]. The maximum emission wavelengths were also all concentrated around 425 nm, indicating that the synthesized CDs had uniform particle sizes and relatively concentrated emission states. This may be due to the various luminescence sites on the surface of CDs or the effect of different CD particle sizes [28]. The quantum yield of 24.1% was determined using quinine sulfate as a reference [29].

### 2.3. Condition Optimization for Rutin Determination by Fluorescent Probe

**Figure 5:**
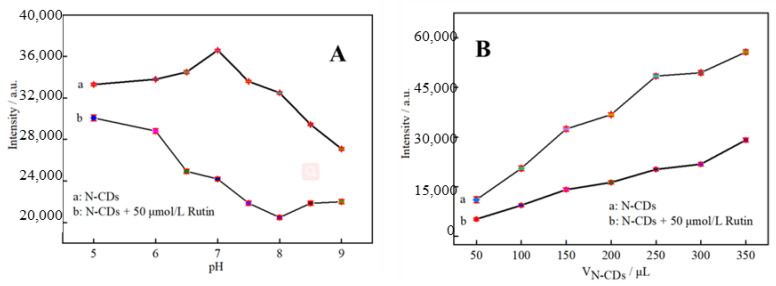
Fluorescence response of N-CDs as a function of pH (**A**) and dosage (**B**) (*n* = 6).

To obtain the optimal conditions for the detection of rutin by theN-CDs fluorescent probe, the effects of the pH of the reaction system and of the amount of N-CDs on the fluorescence intensity were investigated. After 200 μL of N-CDs solution was added into a 10 mL colorimetric tube, the samples were supplemented with 0.1 mol/L PBS in the pH range of 5.0–9.0. The excitation wavelength (E_x_) of 330 nm and emission wavelength (E_m_) of 425 nm were used to measure fluorescence intensity. The same procedure was performed in the presence of 50 μmol/L rutin. The fluorescence intensity of N-CDs varied depending on the pH value, as illustrated in Figure 5A. While the fluorescence intensity of N-CDs in weakly acidic solutions was high and hardly fluctuated, it fell off dramatically when the pH value increased in alkaline matrix solutions. However, 50 μmol/L rutin had a significant and gradually enhanced quenching effect on N-CDs from pH 5 to 8, and the quenching of fluorescence intensity in the presence and absence of rutin was greatest at pH = 7. At pH above 8, the quenching was slightly weakened. Hence, a pH of 7.0 was ideal. This is probably because N-CDs mix with H^+^ to create hydrogen bonds in acidic environments, increasing the system’s robustness. The amino and carbonyl groups on the surface of N-CDs, however, are deprotonated as the pH rises, causing fluorescence quenching and a reduction in fluorescence intensity [30]. 

Subsequently, 50, 100, 150, 200, 250, 300, and 350 μL of N-CDs dispersion was added in sequence into 10 mL colorimetric tubes with and without 50 μL of 0.01 mol/L rutin standard solution. The volume was adjusted with a PBS solution of pH = 7.0, and the results are shown in Figure 5B. In the absence of rutin, the dosage of N-CDs rose along with the fluorescence intensity F_0_. Regardless of how many N-CDs were utilized, rutin had a specific fluorescence-quenching effect on N-CDs, the intensity of which was referred to as F. Due to the abundance of –OH and C–O groups present in both rutin and the N-CDs produced by employing citric acid as the carbon source, strong hydrogen bonds may be formed upon the addition of rutin into the N-CDs solution. It is hypothesized that the fluorescence-quenching process may be triggered by the fluorescence resonance energy transfer and internal filtration effect between N-CDs and rutin after bonding to the surface through hydrogen bonds [31]. Rutin functions as an energy acceptor after fluorescence emission. Calculating F = F_0_ − F, the results revealed that the fluorescence quenching effect was best when 250 μL of N-CDs dispersion was present. N-CDs were, thus, added to the reaction system in a volume of 250 μL.

### 2.4. Performance Analysis

**Figure 6:**
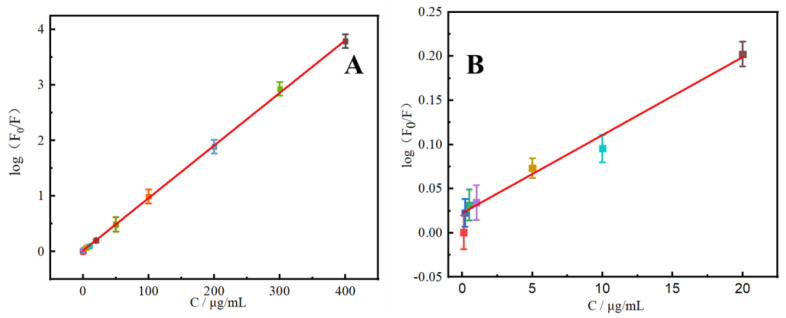
(**A**) Calibration curve of log(F_0_/F) and concentration of rutin (0.1–400 μg/mL). (**B**) The dependence of log(F_0_/F) on the concentrations of rutin within the range of 0–20 μg/mL (*n* = 6).

To evaluate the detection performance of the rutin N-CDs fluorescent probe, the calibration curve was established in a concentration range from 0.1 to 400 μg/mL with log(F_0_/F) = 0.0095c + 0.018 (R^2^ = 0.9996) under the optimal conditions. The detection limit (S/N = 3) was 0.033 μ/mL. Rutin can cause N-CDs in this range to lose fluorescence, as shown in Figure 6. As listed in Table 2, the sensitivity of this analytical technique is comparable to previous CDs fluorescence probe-based rutin detection techniques [32,33,34,35,36]. Rutin is typically found in relatively small amounts in rutin tablets, human serum, human urine, and other biological samples. However, it can be found in varying amounts in dietary samples. The approach described in this work is more suited for the detection of food samples since it has a broad linear range.

### 2.5. Selectivity, Reproducibility, and Stability

**Figure 7:**
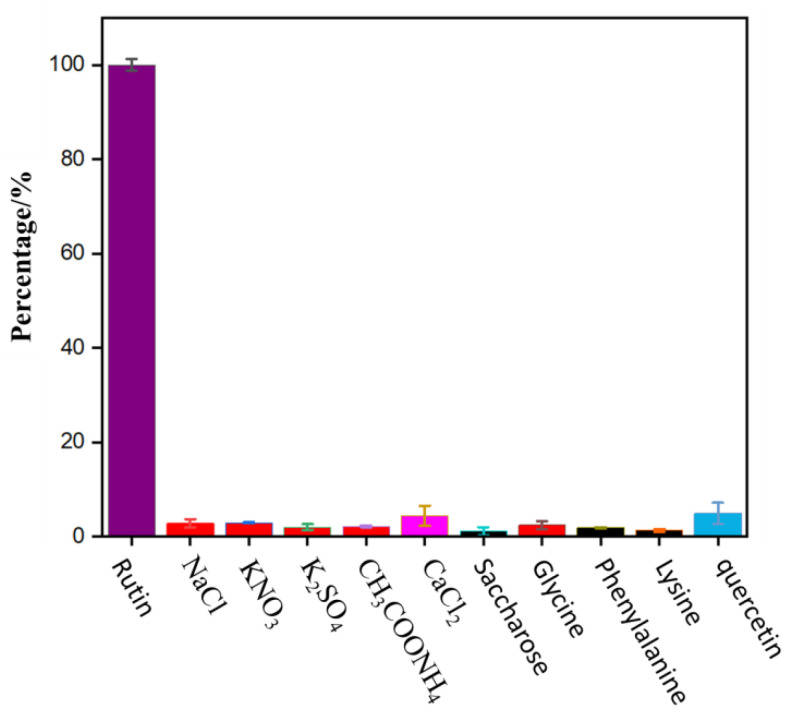
Effects of interfering substances.

To evaluate the selectivity of N-CDs as a fluorescent probe for the detection of rutin, a PBS solution (pH = 7) containing 50 μL of 0.01 mol/L rutin and 250 μL of N-CDs was added along with several external interfering substances that may be present in common foods. As shown in Figure 7, when the relative error was within ±5%, a 100-fold rutin concentration of various salts (NaCl, KNO_3_, K_2_SO_4_, CH_3_COONH_4_, and CaCl_2_), 100-fold rutin concentration of sugar (sucrose), 50-fold rutin concentration of common amino acids (glycine, phenylalanine, and lysine), and five-fold rutin concentration of quercetin did not significantly interfere with the analysis. However, at the same concentration, other flavonoids having similar chemical structure analog, including luteolin, genistein, baicalin, chlorogenic acid, and kaempferol interfered with the determination of rutin significantly. Fortunately, these flavonoids were not found or at a trace level in black buckwheat tea and wolfberry.

The above results indicate that the method has ideal selectivity and a good anti-interference effect. Hence, it can be selectively employed for the determination of rutin.

The reproducibility of the method was evaluated as described below. First, 250 μL of N-CDs were used to detect 50 μmol/L rutin in PBS solution (pH = 7.0) over six successive assays and measured with a relative standard deviation (RSD) of 4.90%. To investigate the stability, the intensity of fluorescence after storage in the refrigerator for 5 days was measured, and then a current value at 94.7% of its initial value was achieved, with an RSD of 2.24% (*n* = 6). These results show that the method in this work has superior reproducibility and stability.

### 2.6. Sample Analysis

Black buckwheat tea and wolfberry samples were tested with rutin standard solution at concentrations of 1, 10, and 100 μg/mL. As indicated in Table 2, under the chosen detection settings, sample recoveries ranged from 93.98% to 104.92%, while relative standard deviations (RSD) ranged from 0.35% to 4.11%. The results indicated that the method used to determine the rutin content in food had high precision and good stability, in line with the requirements of the Chinese National Standard “Criterion on quality control of laboratories chemical testing of food” (GB/T27404-2008). 

In addition, the rutin content of the samples was also determined by high-performance liquid chromatography for comparison (Table 3). The conditions are as following: 

Instrument: Agilent 1260;

Column-Hypersil GOLD C18 250 mm × 4.6 mm × 5.0μm; 

Column temperature: 30 °C; 

Mobile phase: A: methanol, B: 4% formic acid solution, A:B = 40%:60%; 

Flow rate:1.0 mL/min; 

Wavelength: 360 nm;

Injection volume: 10 μL.

The detection results of the two methods were consistent, indicating the high accuracy of the method established in this work.

According to the formula in 2.2.6, the mass percentage of rutin in black Tartary buckwheat tea and Lycium barbarum was 5.34 % and 2.19 %, respectively.

The above results illustrate that the prepared N-CDs fluorescent probe is highly selective for the determination of rutin. Moreover, the standard deviations (*S*) were within the tolerance of the error. The precision, stability, and accuracy of this approach all meet the standard requirements. Therefore, it can be applied to the analysis and detection of actual food samples.

## 3. Materials and Methods

### 3.1. Instruments 

The following instruments were used: F-7000 fluorescence spectrophotometer (Hitachi, Tokyo, Japan), TU-1810 UV/Vis spectrophotometer (Beijing Persee General Instrument Co., Beijing, China), AVATAR-370 Fourier-infrared spectrometer (Thermo Nicolet Corporation, Waltham, MA, USA), Tecnai G2 F20 high-resolution electron transmission microscope (TEM, FEI company, Hillsboro, OR, USA), Octane T Plus X-ray spectrometer (Ametek, San Diego, CA, USA), D8 X-ray diffractometer (XRD, Bruker, Karlsruhe, Germany), ZS 90 nanoparticle size potential analyzer (Malvern, Malvern, UK).

### 3.2. Materials

Citric acid (≥99.5%), thiourea (≥99%), potassium dihydrogen phosphate (≥99%), dipotassium hydrogen phosphate (≥99%), sodium hydroxide (≥98%), orthophosphate (≥85%), ethanol (≥99%), and other pure analytical reagents were procured from Sinopharm Chemical Reagent Co., LTD (Shanghai, China). HPLC-grade rutin standard (≥98%) was obtained from Shanghai Titan Technology Co., LTD (Shanghai, China).. Quercetin, luteolin, genistein, baicalin, chlorogenic acid, and kaempferol standard (≥98%) was obtained from Shanghai Aladdin Technology Co.LTD (Shanghai, China).. Ultrapure water (with a resistivity of 18.2 MΩ·cm) was used in all experiments. 

### 3.3. Preparation of N-CDs

N-CDs were synthesized through a bottom-up approach in the hydrothermal process, following the method in the literature with appropriate adjustment [37]. Briefly, 0.5764 g (0.003 mmol, C_6_H_8_O_7_) of citric acid and 0.2284 g of (0.003 mmol, CH_4_N_2_S) thiourea, as the main carbon and nitrogen sources were weighed and added to 30 mL of ultrapure water in a clean beaker, to obtain a pale, yellow transparent solution, which was dissolved via ultrasonic treatment for 20 min. A clean polytetrafluoroethylene liner was used to transport the solution, which was then put in a reaction kettle at a high temperature. The obtained samples were removed from the reaction after 12 h at 180 °C and allowed to cool naturally to room temperature. The resultant mixture was then filtered using a 0.22 μm filter membrane after being centrifuged at 6000 rpm for 10 min. The overreacted residue, lingering precursors, and byproducts were eliminated by dialyzing the filtered transparent solution (MW = 1000 Da) for 2 days. The transparent N-CDs dispersion was obtained and kept in a dark, 4 °C refrigerator for subsequent use.

### 3.4. Characterization of N-CDs

After drying at 60 °C and then freezing, the N-CDs powder was measured using Fourier-transform infrared spectrometry (FTIR) to identify the functional groups. High-resolution TEM analysis was carried out to evaluate the morphology of N-CDs. The particle size of N-CDs was determined using a nanosize potential analyzer. The X-ray powder diffraction spectrum was obtained by XRD to determine the crystal structure of the products.

### 3.5. Optical Performance Test of N-CDs

After adding 200 μL of N-CDs solution into a 10 mL colorimetric tube, the samples were mixed with 0.1 mol/L phosphate-buffered solution (PBS) at pH = 7.0 and left for 3 min at room temperature. Subsequently, the UV/Vis and fluorescence spectra were recorded.

### 3.6. Sample Pretreatment and Determination of Rutin Content

First, 100 g of black buckwheat tea and 100 g of wolfberry were taken and dried in an oven at 60 °C for more than 5 h. Next, they were taken out and cooled to room temperature and then shattered using a high-speed universal grinder. A certain amount of crushed sample was accurately weighed in a 10 mL centrifuge tube before adding aqueous ethanol (70% *v*/*v*), mixing, shaking, and placing the tube in an ultrasonic cleaner for ultrasonic extraction for 50 min. After removal from the ultrasonic shaker, the tube was centrifuged for 10 min at a speed of 4000 r/min, and the supernatant was filtered through a 0.22 μm microporous filter membrane before testing. Different concentrations (0.1–400 μg/mL) of rutin standard solution were placed in a 10 mL colorimetric tube, followed by the addition of N-CDs solution and PBS solution in turn, before adjusting the volume to the scale and mixing well.

After being left at room temperature for 3 min, an appropriate amount of mixture was put in a quartz cuvette to measure the fluorescence intensity F and the corresponding blank group F_0_ at λ_ex_ = 330 nm and λ_em_ = 425 nm. The mass concentration of rutin C (g/mL) was used as the abscissa of the standard curve, and the corresponding logarithm of the intensity of fluorescence quenching (lg(F_0_/F)) was used as the ordinate.

The mass percentage X (%) of rutin content in the sample to be tested was calculated according to the following equation:

(1) $$X=\frac{c\times V\times K}{m}\times 10^{-4}$$

where *c* is the concentration of rutin (μg/mL) in the solution to be tested, obtained from the standard curve, *V* denotes the volume of the extract added into the sample (mL), *m* is the mass of the sample (g), and *K* is the dilution multiple.

### 3.7. Data Analysis and Processing

Each sample was measured six times in parallel. Excel 2020 and Origin Pro 2021 were used for data processing and chart drawing, respectively.

## 4. Conclusions

As a result of its uniform particle size, good dispersion, abundance of amino, hydroxyl, and carbonyl functional groups on its surface, good water solubility, and stable optical properties, N-CDs, prepared using a one-step hydrothermal method involving citric acid and thiourea, can be used as a fluorescent probe for the detection of rutin content in food. This work demonstrates that rutin can significantly reduce N-CDs fluorescence. Under the ideal detection conditions, the proposed method achieved results in line with the HPLC approach. The rutin detection method based on the fluorescent probe exhibits good sensitivity, precision, stability, and accuracy. Hence, it can be applied for the rapid detection of rutin content in food.

## Figures and Tables

**Table 1:** Element species and content analysis.

Elements	Mass Percentage (%)	Atom Percentage (%)
C	88.46	86.95
N	5.04	4.25
O	5.93	4.37
S	0.57	0.21

**Table 2:** Comparison of rutin detection performance of different CDs as fluorescent probes.

CDs	Linear Range	Detection Limit	Sample	Reference
CDs	0.5–15 (μmol/L)	61 (μg/L)	Drugs	[32]
Cu-CDs	0.1–15 (μg/mL)	0.05 (μg/mL)	Drugs	[33]
T-CDs	0.06–130 (mg/L)	0.02 (mg/L)	Tablets, human serum, human urine	[34]
N-CDs	0.25–10 (μmol/L)	0.15 (μmol/L)	Tablets	[35]
N, S-CDs	0–145 (mg/L)	0.02 (μmol/L)	Lake water Human urine	[37]
N-CDs	0.1–400 (μg/mL)	0.03 (μg/mL)	Black buckwheat tea Wolfberry	This work

**Table 3:** Recovery, precision, and accuracy of determination of rutin in samples (*n* = 6).

Sample	Added (μg/mL)	Detected (μg/mL) ($\overset{-}{x}$ ± *S*)	Recovery (%)	RSD (%)	Detected by T/QAS 013-2020 (μg/mL) ($\overset{-}{x}$ ± *S*)
Black buckwheat tea	0	106.73 ± 0.38	-	0.35	106.84 ± 0.24
	1	107.73 ± 0.38	93.98–103.88	4.11	107.66 ± 0.27
	10	116.71 ± 0.22	96.78–102.51	2.24	116.72 ± 0.21
	100	206.81 ± 2.35	96.22–103.10	2.35	207.11 ± 2.36
Wolfberry	0	43.89 ± 0.67	-	1.53	44.01 ± 0.75
	1	44.89 ± 0.03	97.00–103.83	3.04	44.91 ± 0.03
	10	53.94 ± 0.34	96.71–104.92	3.43	53.99 ± 0.21
	100	144.23 ± 1.95	97.06–102.38	1.95	144.06 ± 1.50

## Data Availability

The data presented in this study are available on request from the corresponding author.
